# Health service providers in Somalia: their readiness to provide malaria case-management

**DOI:** 10.1186/1475-2875-8-100

**Published:** 2009-05-13

**Authors:** Abdisalan M Noor, Ismail A Rage, Bruno Moonen, Robert W Snow

**Affiliations:** 1 Malaria Public Health and Epidemiology Group, Centre for Geographic Medicine, KEMRI – Wellcome Trust Research Programme, Nairobi, Kenya; 2 Centre for Tropical Medicine, Nuffield Department of Clinical Medicine, University of Oxford, CCVTM, Oxford, UK; 3 Centre for International Health and Development Institute of Child Health, University College London, 30 Guilford Street, London WC1N 1EH, UK; 4 The Clinton Foundation, Timau Plaza, Argwings Kodhek Road, 2011, 00100, Nairobi, Kenya

## Abstract

**Background:**

Studies have highlighted the inadequacies of the public health sector in sub-Saharan African countries in providing appropriate malaria case management. The readiness of the public health sector to provide malaria case-management in Somalia, a country where there has been no functioning central government for almost two decades, was investigated.

**Methods:**

Three districts were purposively sampled in each of the two self-declared states of Puntland and Somaliland and the south-central region of Somalia, in April-November 2007. A survey and mapping of all public and private health service providers was undertaken. Information was recorded on services provided, types of anti-malarial drugs used and stock, numbers and qualifications of staff, sources of financial support and presence of malaria diagnostic services, new treatment guidelines and job aides for malaria case-management. All settlements were mapped and a semi-quantitative approach was used to estimate their population size. Distances from settlements to public health services were computed.

**Results:**

There were 45 public health facilities, 227 public health professionals, and 194 private pharmacies for approximately 0.6 million people in the three districts. The median distance to public health facilities was 6 km. 62.3% of public health facilities prescribed the nationally recommended anti-malarial drug and 37.7% prescribed chloroquine as first-line therapy. 66.7% of public facilities did not have in stock the recommended first-line malaria therapy. Diagnosis of malaria using rapid diagnostic tests (RDT) or microscopy was performed routinely in over 90% of the recommended public facilities but only 50% of these had RDT in stock at the time of survey. National treatment guidelines were available in 31.3% of public health facilities recommended by the national strategy. Only 8.8% of the private pharmacies prescribed artesunate plus sulphadoxine/pyrimethamine, while 53.1% prescribed chloroquine as first-line therapy. 31.4% of private pharmacies also provided malaria diagnosis using RDT or microscopy.

**Conclusion:**

Geographic access to public health sector is relatively low and there were major shortages of appropriate guidelines, anti-malarials and diagnostic tests required for appropriate malaria case management. Efforts to strengthen the readiness of the health sector in Somalia to provide malaria case management should improve availability of drugs and diagnostic kits; provide appropriate information and training; and engage and regulate the private sector to scale up malaria control.

## Background

The adoption into policy since 2004 of new artemisinin-based combination treatments (ACT) for uncomplicated malaria has been almost universal across Africa [1]. However, there have been delays in the deployment of these new, efficacious medicines due to concerns about sustained financing, difficulties in integrating revised recommendations into national standard treatment guidelines and drug procurement and distribution to the periphery of the health sector [2-8]. Several studies have highlighted inadequacies of the formal public health sector to guarantee appropriate levels of drugs, training and guidelines to all their service providers several years after the introduction of new ACT drugs [7,9-11]. These studies serve as a reminder that having recommendations for efficacious drugs does not necessarily translate into a health system prepared to deliver these new medicines to target patients.

Somalia revised its national malaria treatment policy in May 2005, abandoning chloroquine as first-line treatment for uncomplicated malaria in favour of a combination of co-administered artesunate and sulphadoxine-pyrimethamine (AS-SP) [12], which was shown to have an adequate clinical and parasitological response by day 28 of between 95% and 99% across three sites in the country [13]. Quinine was recommended as second-line therapy for treatment failures at both hospitals and Mother and Child Health (MCH) facilities [12]. Diagnosis of malaria was to be undertaken using rapid diagnostic tests (RDT) or microscopy at all health facilities except the lower level health posts where the use of clinical diagnosis and presumptive treatment with SP as first-line therapy was recommended. In case of treatment failure with SP at health posts, patients are to be referred to higher-level facilities for treatment [12].

Civil war has ravaged Somalia for over 19 years causing untold suffering, famine and displacement of its population. In 2000, efforts were made by the international community to support the fragile health system and work toward a reconstruction of this sector to support the health needs of Somali's [14]. This coordinated effort received support from multiple international non-government organizations, bi-lateral agencies and support from the Global Fund for AIDS, TB and Malaria (GFATM) [15]. The Somali Aid Coordinating Body, comprised of development partners, UNICEF, WHO and the three ministries of health representing the three regions of Somalia, applied for support from the GFATM during round 2 and were awarded 12.9 million USD in 2002 to implement a new national malaria strategy [15], including the delivery of AS-SP as first-line treatment.

Very little is known about the current capacities of the public and private sector health services in Somalia and we present here the results of a rapid assessment of the extent, coverage and readiness of services able to provide malaria case-management in three districts.

## Methods

### Study sites

**Figure 1:**
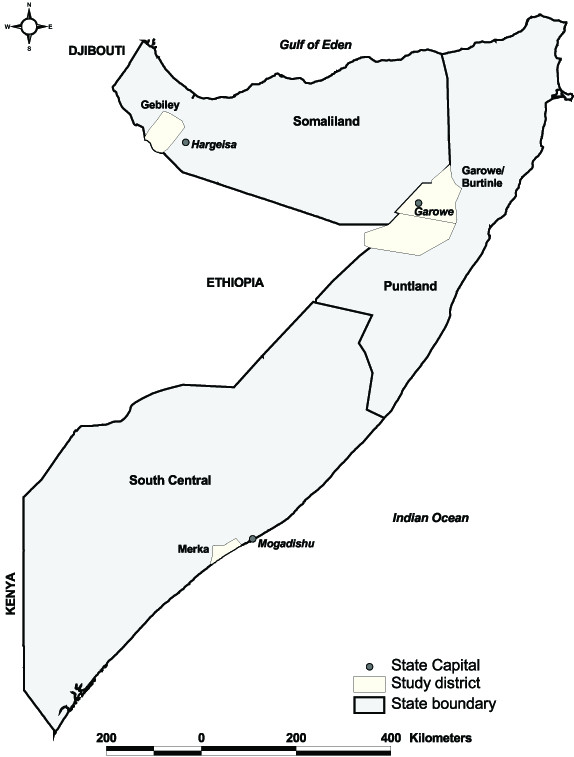
**Map of Somalia showing the two self-declared states (Puntland and Somaliland) and the south-central region; their capital cities; and the study districts**. District boundaries are as defined by the local authorities and may not match internationally recognized boundaries.

Three study districts were purposively sampled in consultation with the respective ministries of health and guided by the security clearance provided by the United Nations from each of the two self-declared republics in Somalia, (Somaliland, Puntland) and the region of south-central Somalia. The districts included Gebiley district, 50 km west of Hargeisa, the capital of Somaliland; Garowe/Burtinle districts in Puntland; and Merka in south central Somalia, bordering the Indian Ocean and 100 km south of Mogadishu (Figure 1). The malaria ecologies vary between these three sites ranging from exceptionally arid and low population density areas in Garowe/Burtinle with low malaria transmission, Gebiley district with similar arid, low malaria transmission conditions but higher human population densities and Merka district with higher malaria transmission located closer to the Shabelle river, more intense agriculture and highest population density [16].

### Health facility and settlement mapping

A baseline digital database of boundaries, settlements, roads and health facilities obtained from the Somalia office of the United Nations Development Programme (UNDP) was used to prepare initial district maps. In collaboration with local health authorities and elders these district maps were further modified to represent recent changes in the boundaries of health service provision for the district. Consultation with district-level health managers provided additional information on known health service providers. Field workers were selected by the respective state ministry of health in collaboration with the WHO local offices and were trained in the administration of survey questionnaires and use of a global positioning system (GPS: Garmin *Etrex*). Field workers were instructed to use the preliminary maps to confirm settlement locations and provisional population sizes and position all health providers managed by NGO's, ministries of health and private sector pharmacies and clinics and record a longitude and latitude for each structure or central position of each settlement. Provisional population totals were obtained from the UNDP settlement data and these were verified using household counts and discussions with village and clan elders. All assembled positional data were later overlaid on Google Earth imagery downloaded from [17] and captured between 2005 and 2007 to triangulate GPS positions.

Each survey team comprised of two interviewers, a driver and a locally appointed guide. In each district a total of five teams were involved in the survey and teams were assigned areas of relatively equal population/area. The survey took place from the 7^th ^– 22^nd ^April 2007 in Gebiley; 25^th ^October – 10^th ^November 2007 in Garowe/Burtinle; and 30^th ^August – 9^th ^September 2007 in Merka.

### Facility audits

Questionnaires were developed to record information on services provided, anti-malarial drugs in stock on the day of the survey, numbers and qualifications of staff, sources of financial support and presence and stock of malaria diagnostic services, new treatment guidelines and job aides for malaria case-management at public health facility. A less detailed questionnaire for private health facilities documenting the type of health facility; malaria services provided and type of drugs used for first- and second-line malaria treatment was also developed. Manuals were developed prior to the survey explaining the questionnaire and providing visual imagery of drugs and supporting case-management aides to assist in training and completion of the service provider audits. At each public and private health facility, the person in-charge at the time of visit was interviewed. Where a facility reported to have a microscope or rapid diagnostic tests (RDT) for malaria, these were confirmed by observation. The availability of trained laboratory technicians, however, was based on interviews of the person in charge of the health facility or pharmacy.

### Data entry and quality control

At the end of the survey all the completed questionnaires were submitted to the local WHO-RBM offices. Each questionnaire was reviewed and inconsistencies were returned to survey teams to reconcile through follow-up visits. All health facility questionnaires data were entered in a customised screen with internal consistency checks developed using EpiData 3 (EpiData Association, Denmark). Spatial coordinates of health facilities and population settlements were imported into ArcGIS 9.1 (ESRI Inc., USA) and overlaid on pre-existing boundary and road maps and Euclidean distances from settlements, excluding the district capitals, to public health facilities were computed.

## Results

### Distribution of service providers and population

**Figure 2:**
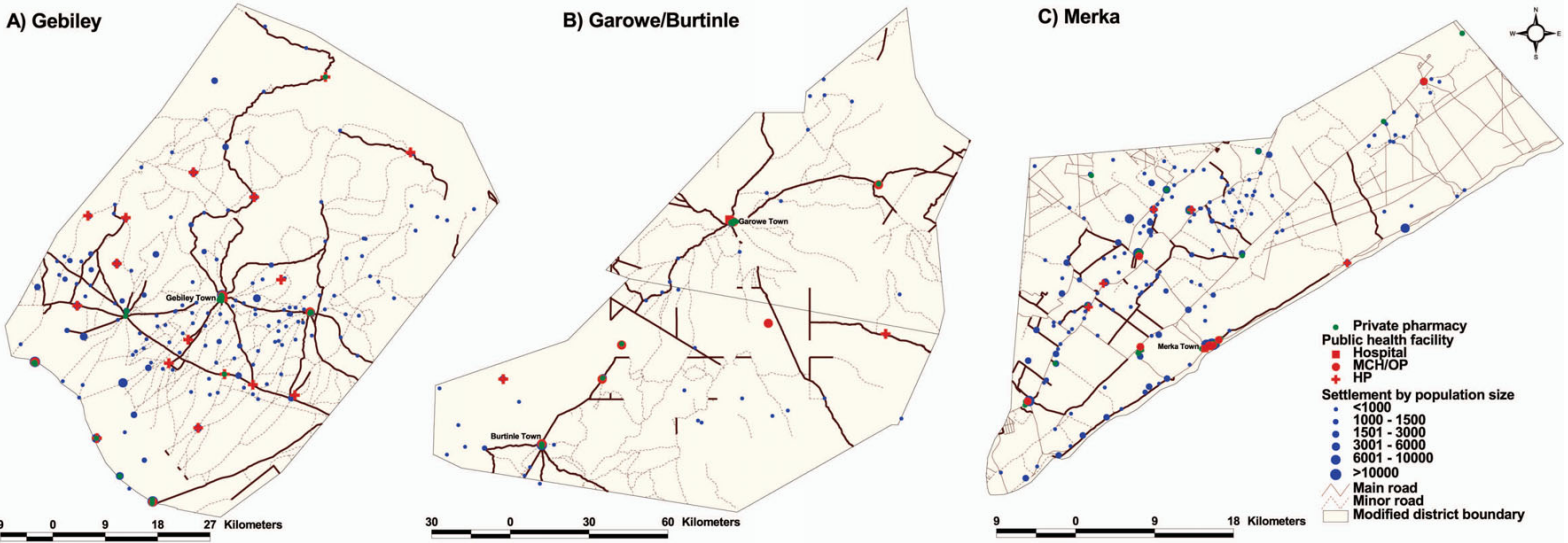
**A-C Maps of study districts showing the location of public and private health facilities; and the distribution of settlements by population size**. District boundaries are as defined by the local authorities and may not match internationally recognized boundaries.

Table 1 shows the numbers of identified health service providers, population settlements and estimated population sizes. Population densities (per Km^2^), population-per-service provider and median distances from settlements outside the capitals of each district to the nearest service provider were computed (Table 1). The distribution of services and population are shown in Figures 2a–c for each district. There were 21 public health service providers in Gebiley district; 10 in Garowe/Burtinle; 14 in Merka district. For each public health service provider there were, on average, 10,952 people in Gebiley; 7,300 in Garowe/Burtinle; 21,429 in Merka; representing an overall ratio of one public health facility for every 11,667 people. The median distances to public health services for settlements excluding the district capitals were highest in Garowe/Burtinle (24.2 km); followed by Gebiley (6.8 km); and the shortest distances to public facilities were found in Merka (4.6 km) (Table 1).

**Table 1:** A description of population density and health service providers in three districts of Somalia in 2007

	**Gebiley** **district**	**Garowe/Burtinle districts**	**Merka district**	**Total**
Area within established district boundary (Km^2^)	4,228.1	19,751.6	1413.5	25,393.2

Number of settlements indentified	163	42	177	382

Estimated total population (population density per Km^2^)	230,000 (54.4)	73,000 (3.7)	300,000 (212.2)	603,000 (23.7)

Total number of public health facilities	**21**	**10**	**14**	**45**
Hospitals	1	1	2	4
MCH/OP facilities	4	7	7	18
Health posts	16	2	5	23

Population per public facility ratio	10,952 per facility	7,300 per facility	21,429 per facility	11,667 per facility

Median (min, max) distance from settlement to public facility outside the district capital (Km)	6.8 (0.1, 16.3)	24.2 (0.1, 45.3)	4.6 (0.1,14.4)	6.3 (0.1, 45.3)

Staff per district				
Doctors	1	5	5	11
Nurses	9	26	35	70
Auxillary Nurses	15	32	44	91
Community Health Workers	15	8	9	32
Laboratory technicians	4	8	11	23

Total number of Private sector providers				
Pharmacy alone	32	36	79	147
Pharmacy + laboratory	15	5	6	26
Pharmacy + clinic	5	1	2	8
Pharmacy +laboratory+ clinic	2	11	0	13

Population per pharmacy ratio	4,260 per pharmacy	1,377 per pharmacy	3,448 per pharmacy	3,108 per pharmacy

MCH/OP = Mother and Child Health/Out-patient facility

Of the 45 public health sector providers across the three districts, four were classified as hospitals providing in-patient care, 18 were second level out-patient providers of MCH services and general out-patient (OP) services, and 23 were classified as health posts that provide out-patient care services against a minimum set of essential drugs and staffed mainly by a single community health worker (Table 1). A total of 227 health personnel were stationed at the 45 health facilities and the majority were nurses (30.8%, n = 70) or auxiliary nurses (40.1%, n = 91). Only 11 qualified doctors were providing clinical services in the public health sector, at the time of the survey serving a total estimated population of 603,000 with only one doctor in Gebiley district. Only five facilities were directly supported by ministries of health, the remaining 40 were supported by NGOs, UNICEF, WHO and through partial cost-recovery. All public health facilities that had a microscope reported to have a trained laboratory technician.

The district mapping exercise identified 194 private health service providers, commonly referred as pharmacies, in the study districts: 54 in Gebiley district; 53 in Garowe/Burtinle district; and 87 in Merka district. Of these, 147 (75.8%) were pharmacies only, seven (3.6%) were pharmacies with an attached clinic, 26 (13.4%) were pharmacies with an attached laboratory but without a clinic, and 13 (6.7%) were pharmacies with both clinic and laboratory (Table 1). Population-per-pharmacy ratios were considerably higher than public sector providers with one pharmacy for every 3,108 people. Considering the pharmacies with clinical services as providers of out-patient care, the private sector contributed approximately 31% of all out-patient care facilities across the three districts.

### Provision of malaria treatment services in the public sector

All public health facilities provided malaria treatment services but only 62.3% (all hospitals and MCH/OPD and seven health posts) reported routinely prescribing the nationally recommended first-line anti-malarial treatment, and 37.8%, of which 88% were in Gebiley district, reported the continued prescription of chloroquine as first-line treatment for malaria (Table 2, Additional File 1). Facilities reporting the adherence to the national first-line therapy were highest in Garowe/Burtinle districts (n = 8, 80.0%) and lowest in Gebiley district where 15/21 (71.4%) of health facilities reported providing chloroquine. Quinine, the second-line therapy for malaria, which is recommended to be used only at hospitals and MCH-OPD facilities, was prescribed in 91% of these facilities in the three districts (Table 2, Additional File 1).

**Table 2:** Combined summaries of anti-malarial services provided by the public health service providers in the three study districts in Somalia

	**Total**		
	**Hospital/MCH-OP (n = 22)**	**HP (n = 23)**	**All (n = 45)**

Reported use of anti-malarials for first-line treatment*			
			
AS-SP	21 (95.5%)	0	21 (46.7%)
SP	0	7 (30.4%)	7 (15.6%)
Chloroquine	1 (4.5%)	16 (69.6%)	17 (37.8%)

Reported use of anti-malarials for second-line treatment**			
			
Never use	1 (4.5%)	14 (63.6%)	15 (33.3%
Quinine	20 (91.0%)	2 (8.7%)	22 (48.9%)
SP	1 (4.5%)	7 (30.4%)	8 (17.8%)

Nationally recommended first-line therapy in stock on day of survey***	12 (54.5%)	3 (13.0%)	15 (33.3%)

Nationally recommended second-line therapy in stock on day of survey	19 (86.4%)	0	19 (42.2%)

Parasitological diagnosis****			
			
None	2 (9.0%)	23 (100.0%)	25 (55.5%)
RDT	20 (91.0%)	0	20 (44.4%)
Microscopy	12 (54.5%)	0	12 (26.7%)
RDT in stock on day of survey	11 (50.0%)	0	11 (24.4%)

Revised national guidelines available at facility****	14 (63.6%)	0	14 (31.1%)

RDT use wall charts available at facility****	18 (81.8%)	0	18 (40.0%)

Anti-malarial dosing wall charts available at facility	18 (81.8%)	1 (4.3%)	19 (42.2%)

Charging for malaria consultation, diagnosis or treatment	8 (36.4%)	9 (39.1%)	17 (37.7%)

HP = health post; MCH/OP = Mother and Child Health/Out-patient facility; SP = sulphadoxine-pyrimethamine; AS-SP = artesunate and SP; RDT = rapid diagnostic test

*National treatment guidelines recommend AS-SP as first-line therapy in hospitals and MCH/OP facilities and SP at health posts.

** National treatment guidelines recommend Quinine as second-line therapy in hospitals and MCH/OP facilities. The guidelines also recommend that health posts refer patients to MCH/OP or hospitals in case of treatment failure with AS-SP.

***First-line drugs were considered out of stock if a hospital or MCH/OP facility did not have either AS or SP or both and if a health post did not have SP on the day of survey.

****National treatment guidelines recommend the use of RDT or microscopy for diagnosis of malaria at hospitals and MCH/OP while at health posts clinical diagnosis and presumptive treatment are recommended.

Only 33.3% of public health facilities had the first-line anti-malarial drug of AS-SP or SP in stock on the day of the survey. Over 86% of hospitals and MCH/OP facilities had quinine in stock for treatment failures (Table 2). Revised national treatment guidelines, developed and distributed in 2006 using funds from GFATM were to be distributed only in the hospitals and MCH/OP facilities but were available in 63.6% of these public health facilities (Table 2). Only 42.2% of facilities had job aides on the new national treatment guidelines in the form of wall charts (Table 2). Parasitological diagnosis, which was not recommended to be used in health posts, was routinely used in 91% of hospitals and MCH/OP facilities with 54.5% reporting the ability to perform microscopy in addition to the use of RDTs (Table 2). However, only 11/22 (50.0%) of hospitals and MCH/OPD facilities had any RDT in stock at the time of survey. Thirty-seven percent of the public health facilities charged for malaria consultation, diagnosis or treatment; 53% of these facilities were in Gebiley district (Additional File 1).

### Private sector provision of malaria treatment

Over 30% of pharmacies provided parasitological diagnostic services (Table 3). 3% of pharmacies in Merka prescribed AS-SP, 9% in Gebiley district and 18% in Garowe/Burtinle district. Between 8% and 37% of district-level pharmacists prescribed SP mono-therapy, with an average across the districts of 9% (Table 3). Up to 53% of private sector providers routinely prescribed chloroquine as the first-line anti-malarial therapy. Cotecxin^®^, an artemisinin mono-therapy, was available in 22 (42%) of pharmacies in Garowe/Burtinle district and 5 (6%) of pharmacies in Merka district, none of the pharmacies in Gebiley district stocked Cotecxin^®^.

**Table 3:** Summary of anti-malarial services provided by the private health service providers in the three study districts in Somalia

	**Gebiley** **n = 54**	**Garowe/Burtinle** **n = 53**	**Merka** **n = 87**	**Total** **n = 194**
Do not provide malaria diagnosis and treatment	4 (8%)	0 (0%)	4 (5%)	8 (4.1%)

Pharmacy stocks and prescribes as first line treatment for malaria				
				
AS-SP	5 (9%)	9 (18%)	3 (3%)	17 (8.8%)
SP	20 (37%)	4 (8%)	12 (14%)	18 (9.3%)
Chloroquine	24 (44%)	18 (34%)	61 (70%)	103 (53.1%)
Quinine	1 (2%)	0 (0%)	2 (2%)	3 (1.5%)
Cotecxin^®^	0 (0%)	22 (42%)	5 (6%)	27 (13.9%)

Provide parasitological services*				
				
RDT	10 (18.5%)	7 (13.2%)	7 (8.0%)	24 (12.3%)
Microscopy	14 (25.9%)	16 (30.2%)	7 (8.0%)	37 (19.1%)

SP = sulphadoxine-pyrimethamine; AS-SP = artesunate and SP; RDT = rapid diagnostic test

*an additional 22 pharmacies which did not have an attached laboratory used rapid diagnostic tests for parasitological diagnosis of malaria

## Discussion

The availability of public health services was relatively low with only 45 public health facilities and 227 health professionals for approximately 0.6 million people and median distance to health facilities of 6 km. This relatively poor access to health facilities is compounded by the generally bad public transport infrastructure in Somalia. All the health posts (n = 23) were manned by community health workers who had received minimal clinical training. Sixty-two percent of public health facilities prescribed the nationally recommended first-line therapy of AS-SP for hospital and MCH/OP and SP for health posts. Eighty-eight percent of the health facilities that did not comply with the national first-line treatment guidelines were in Gebiley district of Somaliland. Importantly several public health facilities in 2007 were still prescribing chloroquine (38%) as first-line therapy. 67% of public facilities that were compliant with the nationally recommended treatment guidelines did not have the first-line drugs in stock on the day of survey. Diagnosis of malaria using RDT or microscopy was performed routinely over 90% of the public health facilities but only half of these had RDT in stock at the time of survey. Malaria related information such as national treatment guidelines; and RDT wall charts were available respectively in between 60% and 80% of the hospitals and MCH/OP facilities but dosing wall charts for AS-SP and SP monotherapy were available in only 42% of the target public health facilities. The private health sector, although four times larger than public sector in numbers of outlets, had only less than 10% prescribing AS-SP as the first-line therapy and the majority prescribed chloroquine (53%) for this purpose. Interestingly over 30% of private pharmacies provided malaria diagnosis using RDT or microscopy.

Although this study represents the first of its kind in Somalia, there are a number of caveats to the results. First, the study was undertaken in only three purposively sampled districts and may not be nationally representative. The reason for adopting a purposive sample was due the logistical and security implications of undertaking such a study nationally in Somalia. The selected districts, however, represent the relatively more prosperous districts in the three study regions and the equivalent health sector indicators are probably only similar, if not poorer, for other districts. Second, information on training received by the private health sector workers was not recorded. This decision was made following advice by the local health authorities that owners of the many, probably unlicensed, private sector providers would interpret as 'police work' any questions on professional qualification and might have adverse security implications for field workers. Third, due to logistical and time constraints, a proper investigation of the performance of health workers in the public sector, in terms of appropriate diagnosis and treatment of malaria, was not undertaken. As a result, of those facilities that used the recommended anti-malarials, the proportion of health workers who prescribed the appropriate dosage was not recorded. Finally, these surveys and audits were only undertaken once in each district during 2007, as such the results represent only a single snap-shot description of the status of service provision in Somalia. However, these audits are useful exercises to guide areas of policy implementation and could serve, assuming a stabilizing of the security situation, as longitudinal sites to measure progress toward national malaria control effort.

The Somalia national malaria strategy has outlined a series of recommendations for malaria case management including the provision of prompt access to appropriate treatment; provision of relevant information to health workers for appropriate case management; the performance of malaria diagnosis using RDT or microscopy at hospitals and MCH/OPD health facilities; and engagement of the informal sector in the provision of these services [12]. On the basis of the acceptance of the new treatments guidelines two-thirds of the public health sector complied but any practical implementation of the policy was hampered by the widespread shortages in drugs and diagnostic tests. There is need to expand the health services to alleviate the long distances people are likely to walk to access health care given the poor state of public transport; establish efficient systems that can overcome the severe shortages of drugs and diagnostic materials; and improve adherence to recommended diagnosis and prescription through in-service training and job aides notably at the peripheral health posts which serve majority of the rural communities. The national malaria strategy should also be revisited to review the continued use of SP at health posts given that this drug has failed and is no longer in use in most African countries.

The service provider audit highlights the prolific nature of the private health sector in Somalia. This scales with recent findings from a national household sample survey of fevers and their treatment, which documented that over 40% of children who had fever in the two weeks prior to survey sought treatment from the private sector compared to only 8% from the public sector [18]. There is a growing recognition internationally of the role of the private sector in the treatment of malaria [19,20]. In Somalia the private sector is perhaps more sophisticated than many other settings in Africa, 30% provided a combination of parasitological diagnosis and anti-malarial treatment. However, as with other settings [21] private sector providers continue to prescribe non-recommended malaria treatments. Ministries of health and stakeholders need to create mechanisms to engage, support and regulate the private sector in Somalia. Population distributions in Somaliland and Puntland converge around main roads and intersections where private health service providers are located for market reasons; the less well-served sparsely distributed and remote rural settlements populations require investments in public health sector provision, as these will still remain the main source of care for these communities.

## Competing interests

The authors declare that they have no competing interests.

## Authors' contributions

AMN was responsible for study design, data cleaning, analysis, interpretation and production of the final manuscript.

IAR contributed to data collection, cleaning and analysis and production of final manuscript.

BM contributed to the design of the questionnaires and data entry platform and was involved in the production of final manuscript.

RWS was responsible for overall scientific management, analysis, interpretation and preparation of the final manuscript.

## Supplementary Material

Additional file 1 **Summary of anti-malarial services provided by the public health service providers in the three study districts in Somalia.** Click here for file
