# Supplementary material for: Health service providers in Somalia: their readiness to provide malaria case-management
Source: Malar J. 2009 May 13;8:100. doi: 10.1186/1475-2875-8-100 (PMC2688519; doi:10.1186/1475-2875-8-100)
Supplement: Additional file 1 — Summary of anti-malarial services provided by the public health service providers in the three study districts in Somalia. [file 1475-2875-8-100-S1.rtf]

Additional File 1 
Summary of anti-malarial services provided by the public health service providers in the three study districts in Somalia
	Gebiley (n=21)	Garowe/Burtinle (n=10)	Merka (n=14)	
	Hospital/
MCH-OP (n=5)	HP
(n=16)	Hospital/
MCH-OP (n=8)	HP
(n=2)	Hospital/
MCH-OP (n=9)	HP (n=5)	
Reported use of anti-malarials for first-line treatment*

AS-SP
SP
Chloroquine	


5 (100.0%)
0 
0 	


0 
1 (6.2%)
15 (93.8%)	


7 (87.5%)
0 
1 (12.5%)	


0 
1 (50.0%)
1 (50.0%)	


9 (100.0%)
0 
0 	


0 
5 (100.0%)
0 
	
Reported use of anti-malarials for second-line treatment**

Never use
Quinine
SP	


0 
5 (100.0%)
0 	


7 (43.8%)
2 (12.5%)
7 (43.8%)	


0 
7 (87.5%)
1 (12.5%)	


2 (100.0%)
0 
0 	


1 (11.1%)
8 (88.9%)
0 	


5 (100.0%)
0 
0 	
Nationally recommended first-line therapy in stock on day of survey***	3 (60.0%)	0 	4 (50.0%)	1 (50.0%)	5 (55.6%)	2 (40.0%)	
Nationally recommended second-line therapy in stock on day of survey	4 (75.0%)	
0 	7 (87.5%)	
0 (0.0%)
	8 (88.9%)	
0 
	
Parasitological diagnosis****

None
RDT
Microscopy
RDT in stock on day of survey	


0 
5 (100.0%)
3 (60.0%)
3 (60.0%)	


16 (100.0%)
0
0
0	


1 (12.5%) 
7 (87.5%)
4 (50.0%)
5 (62.5%)	


2 (100.0%)
0 
0 
0 	


1 (11.1%)
8 (88.9%)
5 (55.6%)
3 (33.3%)	


5 (100.0%)
0 
0 
0 	
Revised national guidelines available at facility****	5 (100.0%)	0 	7 (87.5%)	0 	2 (22.2%)	
0 
	
RDT use wall charts available at facility****
	4 (75.0%)	0 	7 (87.5%)	0 	7 (77.8%)	0 	
Anti-malarial dosing wall charts available at facility
	4 (75.0%)	1(6.2%)	7 (87.5%)	
0 
	7 (77.8%)	0	
Charging  for malaria consultation, diagnosis or treatment	1 (20.0%)	9 (56.3%)	4(50.0%)	0	3 (33.3%)	0	

HP= health post; MCH/OP= Mother and Child Health/Out-patient facility; SP = sulphadoxine-pyrimethamine; AS-SP= artesunate and SP; RDT= rapid diagnostic test

*National treatment guidelines recommend AS-SP as first-line therapy in hospitals and MCH/OP facilities and SP at health posts.

** National treatment guidelines recommend Quinine as second-line therapy in hospitals and MCH/OP facilities. The guidelines also recommend that health posts refer patients to MCH/OP or hospitals in case of treatment failure with AS-SP.

***First-line drugs were considered out of stock if a hospital or MCH/OP facility did not have either AS or SP or both and if a health post did not have SP on the day of survey.

****National treatment guidelines recommend the use of RDT or microscopy for diagnosis of malaria at hospitals and MCH/OP while at health posts clinical diagnosis and presumptive treatment are recommended.
